# Genetic Mapping of Novel Loci Affecting Canine Blood Phenotypes

**DOI:** 10.1371/journal.pone.0145199

**Published:** 2015-12-18

**Authors:** Michelle E. White, Jessica J. Hayward, Tracy Stokol, Adam R. Boyko

**Affiliations:** 1 Department of Biomedical Sciences, College of Veterinary Medicine, Cornell University, Ithaca, New York, United States of America; 2 Department of Population Medicine and Diagnostic Sciences, College of Veterinary Medicine, Cornell University, Ithaca, New York, United States of America; Texas A&M University, UNITED STATES

## Abstract

Since the publication of the dog genome and the construction of high-quality genome-wide SNP arrays, thousands of dogs have been genotyped for disease studies. For many of these dogs, additional clinical phenotypes are available, such as hematological and clinical chemistry results collected during routine veterinary care. Little is known about the genetic basis of variation in blood phenotypes, but this variation may play an important role in the etiology and progression of many diseases. From a cohort of dogs that had been previously genotyped on a semi-custom Illumina CanineHD array for various genome-wide association studies (GWAS) at Cornell University Hospital for Animals, we chose 353 clinically healthy, adult dogs for our analysis of clinical pathologic test results (14 hematological tests and 25 clinical chemistry tests). After correcting for age, body weight and sex, genetic associations were identified for amylase, segmented neutrophils, urea nitrogen, glucose, and mean corpuscular hemoglobin. Additionally, a strong genetic association (*P* = 8.1×10^−13^) was evident between a region of canine chromosome 13 (CFA13) and alanine aminotransferase (ALT), explaining 23% of the variation in ALT levels. This region of CFA13 encompasses the *GPT* gene that encodes the transferase. Dogs homozygous for the derived allele exhibit lower ALT activity, making increased ALT activity a less useful marker of hepatic injury in these individuals. Overall, these associations provide a roadmap for identifying causal variants that could improve interpretation of clinical blood tests and understanding of genetic risk factors associated with diseases such as canine diabetes and anemia, and demonstrate the utility of holistic phenotyping of dogs genotyped for disease mapping studies.

## Introduction

Numbers of genome-wide association studies (GWAS) in humans and other organisms have increased rapidly as dense single-nucleotide polymorphism (SNP) arrays have become more common and cost-effective and as improved genome annotation and statistical methods have increased the power of these studies[1]. In dogs, these studies have focused on identifying causal variants and risk factors for genetic disease as well as mutations underlying morphological traits like body size and coat color[2]. In contrast, little work has been done to identify variants affecting normal clinical phenotypes, such as those measured by hematological and blood chemistry tests. These blood phenotypes do not represent case/control endpoints but may have a substantial influence on disease susceptibility. Importantly, disease status alone can mask underlying heterogeneity in the pathways leading to genetic diseases, so intermediate phenotypes may offer higher power for genetic mapping and improved understanding of genetic pathways underlying common diseases.

In humans, genetic associations with blood phenotypes have improved the understanding and treatment responses of diverse diseases, including coronary artery disease (CAD) and cancer. For example, a SNP in *HMGCR*, the gene encoding hydroxyl-3-methylglutaryl coenzyme A reductase, was associated with lower levels of low density lipoprotein cholesterol and lower CAD risk[3]. This finding was consistent with the observed reduction in CAD risk with statins, drugs that inhibit the protein product of *HMGCR*. Discovery of the parallel mechanisms behind the disease-associated SNP’s effect on low density lipoprotein cholesterol levels and subsequent disease and the effects of cholesterol-lowering statins on the same target helped focus research efforts on SNPs affecting other blood lipids to determine if additional genetic associations with reduced CAD risk may lead to the development of novel therapeutic agents[3]. Genome-wide association studies of metabolic traits have also led to important links between genetics and drug sensitivities, including an association between androgen metabolism and the *Aldo-Keto Reductase Family 1*, *Member C1* (*AKR1C*) gene that strongly impacts the efficacy and side effects of chemotherapeutic drugs that inhibit *AKR1C*[4]. Understanding the metabolic basis of drug reactions can provide critical knowledge for the development of drugs with decreased risk of toxicities or other complications.

Genome-wide association studies of blood phenotypes in dogs have not yet been conducted, although variations in many blood phenotypes likely have a strong genetic basis. Principal component analysis of 12 hematological parameters from 6000 dogs (75 breeds) revealed distinctive hematological signatures in half the breeds suggesting a significant contribution from genetic loci to differences in these phenotypes[5]. Breed-specific reference intervals are recommended for some hematological phenotypes[6], although genetic mapping is needed to determine the variants contributing to these differences, and possibly establishing more precise reference intervals for individual dogs on the basis of their genetic profiles.

Recently, a collection of over 4,200 dogs was genotyped on a dense SNP array platform for genome-wide analysis of canine demographic history as well as trait and disease association studies[7,8]. This enabled identification of several loci of large effect size that were significantly associated with morphological traits including body size and fur characteristics as well as several loci significantly associated with risk of diverse diseases including hip dysplasia, epilepsy, and lymphoma. Many of these samples were from patients at the Cornell University Hospital for Animals (CUHA), enabling access to medical histories and laboratory data for these dogs. During the course of treatment, many of these dogs had undergone complete blood count (CBC) and clinical chemistry panel (CCP) tests. In total, 353 healthy adult dogs with 39 hematological and clinical chemistry tests were genotyped on a dense 180,000 marker SNP array, enabling the first ever comprehensive mapping study of canine blood phenotypes.

## Materials and Methods

### Samples

All blood samples for CBC and CCP tests were collected at CUHA as part of routine veterinary care with owner consent. Whole blood was collected from the jugular, cephalic or lateral saphenous vein into vacutainers containing EDTA (CBC) and no anticoagulant or heparin anticoagulant (CCP) and analyzed within 48 hours of collection in the Clinical Pathology laboratory at Animal Health and Diagnostic Center (AHDC) at Cornell University. For the CBC, EDTA-anticoagulated blood was analyzed with an automated hematology analyzer, the ADVIA 2120 (Siemens Healthcare Diagnostics Inc., Tarrytown, NJ) using manufacturer’s reagents and a canine-specific setting. The analyzer provided results for red blood cells (count, hemoglobin concentration, hematocrit, mean cell volume [MCV], mean corpuscular hemoglobin [MCH], mean corpuscular hemoglobin concentration [MCHC], red blood cell distribution width [RDW]), white blood cells (total count) and platelets (count, mean platelet volume). A leukocyte differential count (100 leukocytes) was performed by trained medical or veterinary technologists on Wright’s-stained blood smears and yielded individual leukocyte percentages (segmented neutrophils, lymphocytes, monocytes, eosinophils and basophils). The percentages were multiplied by the total leukocyte count from the analyzer to yield absolute individual leukocyte counts. Non- or heparin anticoagulated tubes were centrifuged at 3000*xg* for 10 minutes at 20–23°C. A routine small animal clinical chemistry panel was performed on the separated serum or heparinized plasma with an automated wet chemistry analyzer (Modular P Chemistry Analyzer, Roche Diagnostics, Indianapolis, IN) using manufacturer’s reagents.

With owners’ consent, we used the CUHA medical records database from 2007–2014 to identify dogs genotyped in Hayward, *et al*.[7] that had concurrent CBC and CCP results and were between the ages of 1.5 and 15 years old. Each dog received a complete physical examination from a licensed veterinarian during each visit. These findings, along with the medical history and subsequent test results for each dog were added to the patient’s records. To identify healthy dogs for inclusion in the study, the medical records of these 1,084 dogs were analyzed for age, disease diagnosis, and prescribed medications. Results were excluded from dogs on medications known to alter CBC or CCP parameters (e.g., corticosteroids[9], chemotherapeutic agents[10]) or with systemic illnesses (e.g., diabetes mellitus, hyperadrenocorticism, liver disease, renal disease, cancer). The remaining test results were then screened for outlier values (S1 Table) and principal component analysis (PCA) was performed to identify individuals clustering based on diagnoses (e.g., fracture) or specific phenotypes (e.g., high amylase activity) that likely reflect an underlying disease process. After removal of these outliers, 353 dogs remained in the dataset (285 with both CBC and CCP data, 18 with only CBC, and 50 with only CCP) and no further outliers or strong clusters were observed in PCA. Some of these dogs were presented as potential healthy controls for screening by the Medical Genetics service, though most dogs presented for a medical complaint that did not substantially alter blood phenotypes (e.g., rupture of the cranial cruciate ligament, corneal ulcer, benign skin or subcutaneous mass) or for a routine follow-up after recovery from a previous illness. These remaining 353 dogs were considered clinically healthy for the purposes of this study. For some dogs, panels of a given type from multiple visits remained and were included in the linear mixed model as a repeated measure from the same individual (CBCs per dog: average = 1.5, max = 8; CCPs per dog: average = 1.3, max = 6).

### Genetic analysis

To investigate and control for non-genetic factors that may influence blood phenotypes and reduce the power of genetic association studies, we constructed a repeated measures mixed effects model using the R package lmer[11,12]. Individual variation was treated as a random effect, and spay/neuter status was considered a fixed effect, along with sex, age and ln(body weight). Where appropriate, blood phenotypes were transformed (ln or square root transformations) to normalize the data and residuals. To improve the accuracy of the mixed effect estimates, we included in the mixed effects model an additional 848 healthy adult dogs with CBC and CCP data collected during routine orthopedic or dental evaluations at CUHA but for which genotypic data were lacking. Residual values for each genotyped dog were used as the phenotypes in the genetic association analyses.

Genome-wide association studies were run using a linear-mixed model in GEMMA version 0.94[13], which calculates a kinship matrix across all individuals and includes this as a random effect. Only SNPs with a minor allele frequency (MAF) > 0.05 were included. Bonferroni correction (α = 0.05) based on the 145,719 SNPs remaining after quality control and filtering resulted in a significance threshold for quantitative trait loci (QTLs) of 3.43 ×10^−7^. Manhattan and QQ plots were generated in R version 3.1.2, and linkage disequilibrium (LD) plots were generated using matplotlib library[14] in ipython notebook[15].

## Results

**Fig 1:**
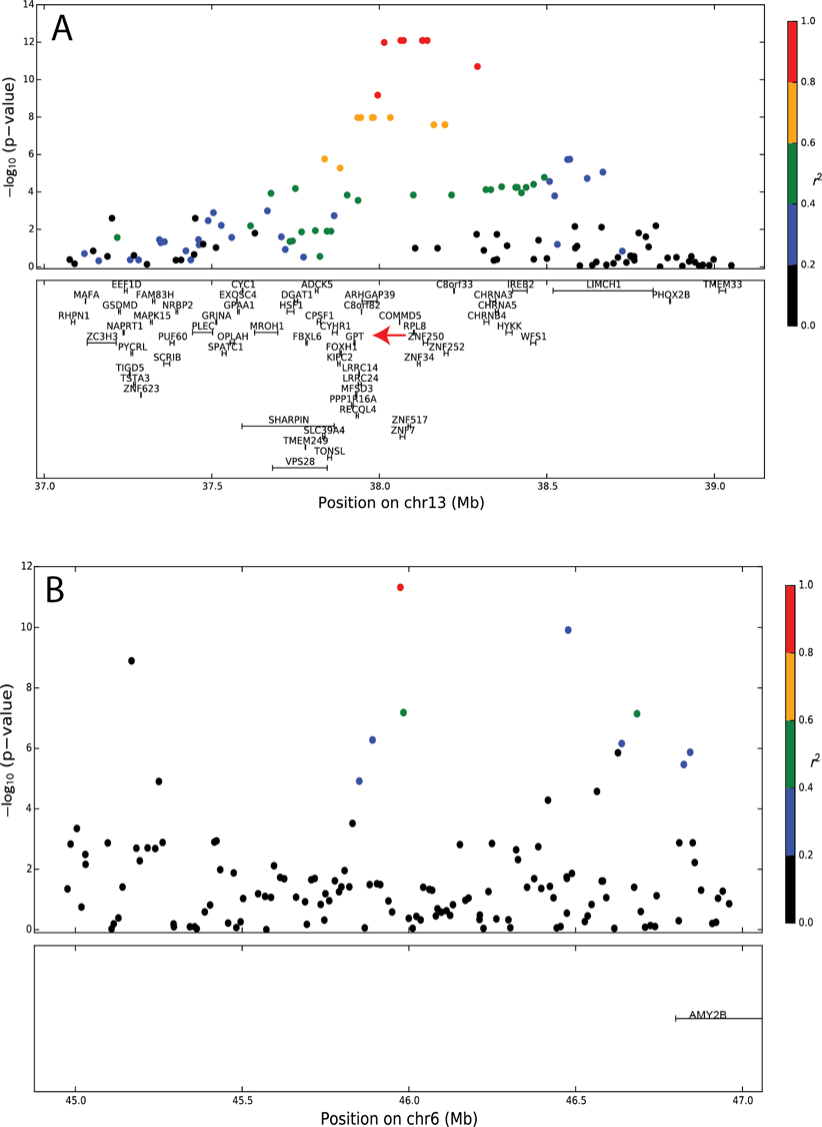
Fine-scale patterns of association at ALT and amylase QTLs. **–**log_10_P values of single marker associations with (A) ALT and (B) amylase activity. Circle color indicates level of linkage disequilibrium (r^2^) between each marker and the most associated SNP. The bottom panels show the relative position of genes in the region. Coordinates for the AMY2B gene from Axelsson, *et al*. (2013)[16].

**Fig 2:**
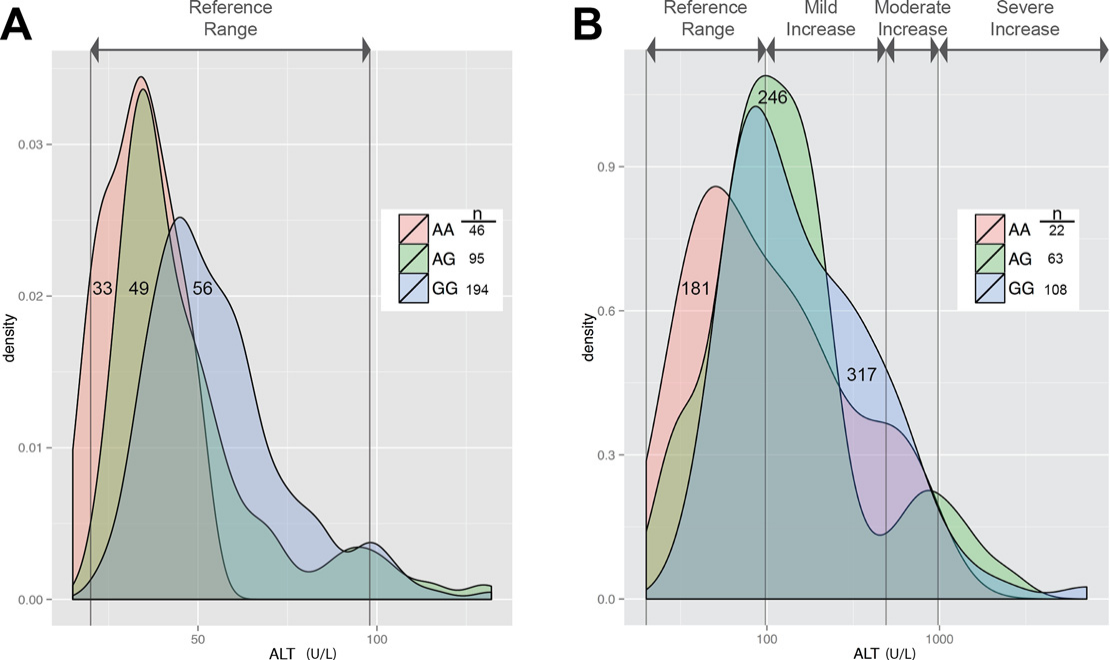
ALT activity by genotype. Distribution of ALT activity by genotype of the most highly associated SNP on chromosome 13 in (A) healthy dogs and (B) dogs with liver disease or injury. Reference intervals and ranges for ALT activity defined as mild, moderate, and severe increases[17] are shown with gray lines. Mean ALT activity is indicated on each distribution. Note the logarithmic scale of the x-axis in panel (B). In both healthy dogs and dogs with liver pathology, the A allele is associated with lower activity of ALT.

Nine of the 39 blood hematological and clinical chemistry test phenotypes yielded significant genetic associations after Bonferroni correction (Table 1, Fig 1 and S1–S5 Figs). The strongest genetic association (*P* = 8.09×10^−13^) was observed between alanine aminotransferase (ALT) activity and several SNPs within a 300kb region on chromosome (CFA) 13 containing nearly a dozen genes (Fig 1A). Notably, the *Glutamic-Pyruvate Transaminase (Alanine Aminotransferase)* gene, *GPT*, in this region encodes the transferase, making it a strong candidate gene driving the association. The derived haplotype, at 28% frequency in our cohort, was significantly associated with lower ALT activity, with heterozygous and homozygous dogs showing a 14% and 42% reduction in ALT activity, respectively, compared to dogs without the variant (Fig 2A).

**Table 1:** Genetic loci with significant associations for canine blood phenotypes. Arranged in order of association significance. Positions in CanFam3 assembly.

Chromosome	Position	MAF	Phenotype	*P* value	Candidate Gene(s)
CFA13	37978745–38292816	0.28	ALT	8.09 × 10^−13^	GPT
CFA6	45168371–46684000	0.14	Amylase	5.12 × 10^−12^	AMY2B
CFA37	25779699	0.08	MCH	2.71 × 10^−8^	ATG9A, ABCB6
CFA16	36539674	0.26	Segmented Neutrophils	8.45 × 10^−8^	DLC1
CFA5	70157846	0.20	Urea Nitrogen	8.96 × 10^−8^	PLCG2
CFA5	62375943	0.23	Globulin	1.48 × 10^−7^	-
CFA17	15481643	0.08	Monocytes	1.68 × 10^−7^	HS1BP3
CFA5	66453261	0.10	Glucose	2.32 × 10^−7^	FOXC2
CFA4	85582167	0.06	MCHC	3.17 × 10^−7^	-
CFA5	65289598	0.10	Monocytes	3.40 × 10^−7^	-

Because ALT activity is commonly used as a marker of hepatocellular injury in humans and dogs, we sought to determine if this association was still evident in dogs with defined liver disease. We re-examined the original genotyped dataset for dogs whose results had been excluded because of a liver disease diagnosis in the medical record (e.g., hepatitis, portosystemic shunts) or dogs with elevated levels of aspartate aminotransferase (AST), an additional enzyme used as a marker of liver injury. Since AST is also found in muscle and is also a marker of muscle injury, dogs with high creatine kinase (CK) activity, a specific marker of muscle injury, were excluded from this additional analysis. The 193 dogs with liver disease or injury (66 diagnosed, 127 based on elevated AST values) showed a similarly strong association between ALT activity and the GPT locus (22% and 43% reduction in heterozygous and homozygous dogs, respectively), with a greater proportion of homozygous dogs with liver injury having ALT activity within the reference interval compared to heterozygous or wildtype dogs (Fig 2B, Table 2). In contrast, the activity of AST was not significantly associated with this locus in healthy dogs, and slightly positively associated with the derived haplotype in sick dogs, suggesting possibly that moderately sick dogs were less likely to be diagnosed with liver damage if they carried the derived haplotype (S6 Fig, S3 Table).

**Table 2:** Correlation of aspartate aminotransferase (AST) activity and *GPT* genotype with alanine aminotransferase (ALT) activity in clinically healthy dogs and dogs with liver disease or injury. *GPT* SNP genotypes were assigned a numerical value (0 = G/G, 1 = A/G, 2 = A/A). Ln(ALT) activity was positively correlated with ln(AST) activity and was negatively correlated with the number of derived A *GPT* alleles for both clinically healthy dogs and dogs with liver disease or injury.

		ln(AST)		*GPT* Genotype	
	N	Effect Size	*P* value	Effect Size	*P* value
**Clinically healthy**	**330**	**0.4**	**6.4x10** ^**-8**^	**-0.23**	**<2x10** ^**-16**^
**Liver disease or injury**	**193**	**0.98**	**<2x10** ^**-16**^	**-0.28**	**1.8x10** ^**-4**^

**Fig 3:**
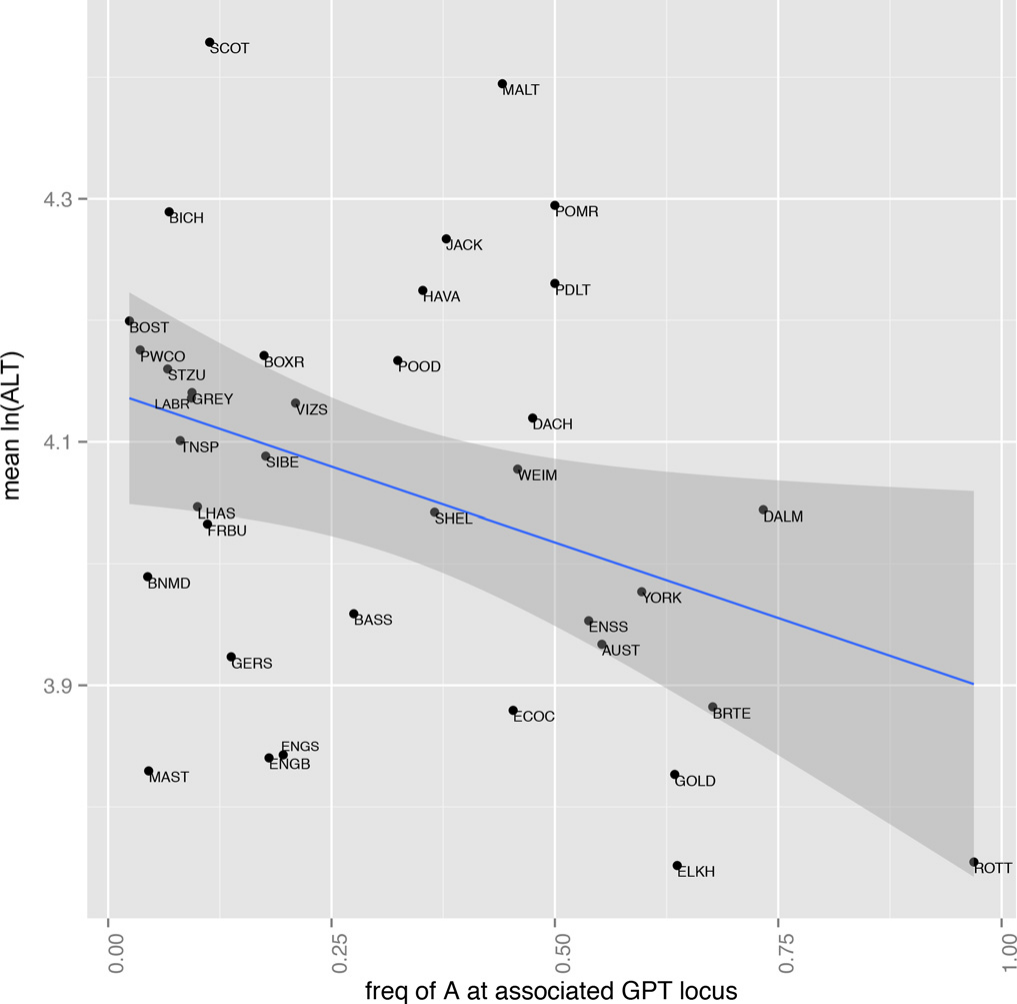
Association between ALT activity and *GPT* allele frequency by breed. Mean ln(ALT) activity for breeds (N≥20 dogs per breed) plotted by derived allele frequency for the *GPT* locus. Frequency of 100% would correspond to GPT locus fixed for A allele (AA genotype). See S2 Table for full breed names and mean ln(ALT).

To determine if our identified novel association between the CFA13 locus and ALT activity was associated with specific breeds, we retrieved five years of ALT results (12,145 unique dogs, N≥20 per breed) within our accepted range (see S1 Table) from ungenotyped dogs that belonged to 44 breeds for which we did have sufficient genotyping data (2,888 unique dogs, N≥14 per breed) in the Cornell Veterinary Biobank[18] to assign allele frequencies for the GPT locus. Although the genotype data and ALT data come from different sets of dogs, we find a significant correlation between mean ln(ALT) by breed versus the allele frequency for the CFA13 locus (*P* < 0.034) with breed-average ln(ALT) decreasing with increasing frequency of the derived allele (Fig 3).

The next most significant association (*P* = 5.21×10^−12^, Table 1) occurred between amylase activity and a ~2Mb region of CFA6 containing the amylase gene *Alpha-amylase 2B* (*AMY2B*, Fig 1B). The gene *AMY2B* harbors an 8-kb copy number variable region that has been previously associated with both amylase mRNA expression levels and amylase activity in serum[16,19]. Although our array did not directly assay the highly variable copy number variant (2–30 copies have been observed in individual dogs), five SNPs in this region were significantly associated with amylase activity in our study, suggesting multiple SNPs are in linkage with the *AMY2B* copy-number genotypes and that this locus explains at least 11% of the variation in blood amylase activity in our dataset.

Several other blood hematological and clinical chemistry phenotypes were significantly associated with loci containing promising candidate genes worthy of further investigation. The region of CFA37 significantly associated (*P* = 2.71×10^−8^) with MCH (S1A Fig) contains *ATG9A*[20] whose product is a component of the interactome for red blood cell assembly and organization, as well as *ABCB6* that encodes a protein with a known role in red blood cell nucleic acid metabolism, protein trafficking, and small molecule biochemistry[20]. Segmented neutrophil count was significantly associated (*P* = 8.45×10^−8^) with a SNP within the gene *DLC1 Rho GTPase activating protein* (*DLC1*, S1B Fig). The dynein motor complex, encoded by *DLC1* and its partner gene *DLC2*, is involved in binding to pro-apoptotic Bim protein, a member of the Bcl-2 family[21]. Changes in these interactions affect the survival time of neutrophils[22]. Monocyte concentration was significantly associated with two loci (*P* = 1.68×10^−7^ and *P* = 3.4 ×10^−7^, respectively), the most significant of which was on CFA17 near the *hematopoietic-specific protein 1 binding protein 3* (*HS1BP3*, S1C Fig) gene, that has been implicated in lymphocyte activation[23]. Monocytes, as antigen-presenting cells, influence lymphocyte activation[24]. Activated lymphocytes, in turn, can produce cytokines that influence monocyte production or trafficking, such as granulocyte-monocyte colony-stimulating factor and interferon-**α**[25]. The gene *FOXC2* is located near the SNP on CFA5, which was significantly associated (*P* = 2.32×10^−7^) with glucose concentration (S1D Fig). FOXC2 is a forkhead transcription factor associated with insulin resistance and regulation of metabolism in humans, for whom expression of *FOXC2* increases in response to insulin and high-calorie diets[26].

Although just outside the range of significance (*P* = 5.90×10^−7^), the association of lymphocyte concentration with a SNP on CFA19 is noteworthy because of the close proximity of the SNP to candidate gene *Alpha-1*, *6-Mannosyl-Glycoprotein Beta-1*, *6-N-Acetyl-Glucosaminyltransferase* (*MGAT5*, S1E Fig). MGAT5 is involved in negatively regulating T-cell activation and autoimmunity[27] and T cells are the dominant lymphocyte population in canine blood[28].

## Discussion

We have shown that we can detect significant genetic associations with 9 blood hematological and clinical chemistry test phenotypes using just 353 canine samples. Although our group of associations is dwarfed by the large number of blood associations detected from GWAS of inbred mouse strains[29] and humans[30], it is noteworthy that such strong associations can be detected in dogs using relatively small sample sizes. For amylase and ALT activity, a sizeable portion (10–20%) of the variation in the phenotype is explained by a single QTL. These large effect sizes are consistent with the simplified genetic architecture of complex morphological traits in dogs, suggesting that for at least some blood phenotypes, selection for related phenotypes or perhaps drift may have led to high frequency genetic variants with a large effect size.

One of the major factors limiting the utility of genotyping for trait or disease-associated SNPs for screening and diagnostic purposes in human medicine has been the predominance of loci with small effect sizes[31]. Our discovery of large-effect loci affecting some CBC and CCP phenotypes suggests a greater utility for direct genotyping of these SNPs in dogs as part of an accurate interpretation of the clinical status of individual patients. Specifically, we believe that directly genotyping the locus associated with ALT activity may be useful in the diagnosis and subsequent treatment of liver disease and injury in the dog, particularly if the causal variant within the 300kb association interval can be identified. Dogs with one or more copies of the minor allele associated with lower ALT activity may be more likely to have liver injury that goes undiagnosed and/or untreated based on standard blood testing. The identification of a high frequency of this allele in specific breeds adds to known breed-specific differences in certain hematological and CCP phenotypes that warrants establishment of more specific reference intervals for certain groups of dogs. Though breed-specific reference intervals are an option to improve on current practices, reference intervals based on genetic profiles would be more precise.

Similarly, direct genotyping of *AMY2B* in dogs may be useful in preventing and managing obesity. An inverse correlation has been observed between copy number of the salivary amylase gene *AMY1* and body mass index (BMI) in humans. Humans with more copies of the amylase genes *AMY1* have higher amylase activity but reduced risk of obesity, in part due to differential starch digestion and potentially increased insulin sensitivity[32]. A similar situation may also occur in dogs with *AMY2B* copy number variation[19] although more studies are needed to confirm the link.

Even when effect sizes are modest, identifying genes involved in hematological phenotypes through GWAS can be highly useful for identifying opportunities for personalized medicine during diagnosis and treatment. Recent GWAS in humans have identified many genes involved in the numbers and characteristics of red blood cells[33], lipids[3], and other metabolites[30] that can be clinically useful both in predicting disease risk and responses to drug regimens (including potential side effects). Additionally, a recent GWAS for blood cell phenotypes in mice identified four SNPs influencing red blood cell phenotypes with the same candidate genes published in the human GWAS literature, as well as six loci associated with white blood cell results currently identified only in mice[29]. Further mapping studies in dogs are needed to determine the degree to which homologous genes or pathways underlie variation in hematological phenotypes in humans, mice and dogs.

The ability to more effectively prevent, diagnose, and treat complex diseases through personalized medicine depends on improved understanding of genetic variation and the interactions between genotypes and other factors influencing disease risk and phenotypes[34]. Routine clinical pathologic blood tests, including CBC and CCP tests, provide standardized measurements of red and white blood cells, platelets, enzymes, and metabolites, many of which are tightly regulated by the body in health to maintain homeostasis[5]. Consideration of these measurements in comparison to established reference intervals is often a crucial step in identifying underlying disease and for choosing further diagnostic tests and treatments as well as monitoring treatment responses in human and veterinary medicine. Further work identifying causal variants underlying genetic associations with canine blood phenotypes may enable personalized reference intervals to be calculated from genetic profiles and identify genotype-specific changes to CBC and CCP parameters during disease progression[35]. Ultimately, understanding the genetic basis of variation in these key clinical phenotypes will be crucial for a deeper understanding of the interplay between canine genetics and disease that will inform both veterinary diagnostics and translational research in personalized medicine.

## Supporting Information

S1 Fig Fine-scale patterns of association at select QTLs. –log_10_P values of single marker associations with (A) Mean Corpuscular Hemoglobin, (B) Segmented Neutrophil Count, (C) Monocyte Count, (D) Glucose and (E) Lymphocyte Count. Circle color indicates level of linkage disequilibrium (r^2^) between each marker and the most associated SNP. The bottom panels show the relative position genes in the region.
(TIF)

S2 Fig GWAS results for Complete Blood Count (CBC) Phenotypes. -log_10_P values of association between each SNP and each (transformed) phenotype. 1. Hematocrit, 2. Hemoglobin, 3. Red Blood Cell Count, 4. Mean Corpuscular Volume (MCV), 5. Mean Corpuscular Hemoglobin (MCH), 6. Mean Corpuscular Hemoglobin Concentration (MCHC), 7. Red Cell Distribution Width (RDW), 8. White Blood Cell Count, 9. Segmented Neutrophil Count, 10. (Square Root) Lymphocyte Count, 11. (Square Root) Monocyte Count, 12. (Square Root) Eosinophil Count 13. (Square Root) Platelet Count, 14. Mean Platelet Volume.
(TIF)

S3 Fig GWAS results for Clinical Chemistry Profile (CCP) Phenotypes. log_10_P values of association between each SNP and each (transformed) phenotype. 1. Sodium, 2. Potassium, 3. Chloride, 4. Bicarbonate, 5. Anion Gap, 6. Sodium to Potassium Ratio, 7. (Ln) Urea Nitrogen, 8. Creatinine, 9. Calcium, 10. (Square Root) Phosphate, 11. Magnesium, 12. Total Protein, 13. Albumin, 14. (Ln) Globulin, 15. Albumin to Globulin Ratio, 16. (Square Root) Glucose, 17. (Ln) Alanine Aminotransferase (ALT), 18. (Ln) Aspartate Aminotransferase (AST), 19. Alkaline Phosphatase, 20. (Square Root) Amylase, 21. Cholesterol, 22. (Ln) Creatine Kinase (CK), 23. Iron 24. Total Iron Binding Capacity, 25. (Ln) Percentage Transferrin Saturation (Saturation).
(TIF)

S4 Fig Quantile-quantile plots by Complete Blood Count (CBC). Quantile-quantile plots of observed versus expected association P-values. 1. Hematocrit, 2. Hemoglobin, 3. Red Blood Cell Count, 4. Mean Corpuscular Volume (MCV), 5. Mean Corpuscular Hemoglobin (MCH), 6. Mean Corpuscular Hemoglobin Concentration (MCHC), 7. Red Blood Cell Distribution Width (RDW), 8. White Blood Cell Count, 9. Segmented Neutrophils, 10. (Square Root) Lymphocyte Count, 11. (Square Root) Monocyte Count, 12. (Square Root) Eosinophil Count, 13. (Square Root) Platelet Count, 14. Mean Platelet Volume.
(TIF)

S5 Fig Quantile-quantile plots by Clinical Chemistry Profile (CCP) Phenotype. Quantile-quantile plots of observed versus expected association P- values. 1. Sodium, 2. Potassium, 3. Chloride, 4. Bicarbonate, 5. Anion Gap, 6. Sodium to Potassium Ratio, 7. Urea Nitrogen, 8. Creatinine, 9. Calcium, 10. (Ln) Phosphate, 11. (Square Root) Magnesium, 12. Total Protein, 13. Albumin, 14. (Ln) Globulin, 15. Albumin to Globulin Ratio, 16. (Square Root) Glucose, 17. (Ln) Alanine Aminotransferase (ALT), 18. (Ln) Aspartate Aminotransferase (AST), 19. Alkaline Phosphatase, 20. (Square Root) Amylase, 21. (Square root) Cholesterol, 22. (Ln) Creatine Kinase (CK), 23. (Square root) Iron 24. Total Iron Binding Capacity, 25. (Ln) Percentage Transferrin Saturation (Saturation).
(TIF)

S6 Fig AST activity by genotype. Distribution of AST activity by genotype of the most highly associated SNP for ALT on chromosome 13 in (A) healthy dogs and (B) dogs with liver disease or injury. Reference interval for AST activity shown with gray lines. Mean AST activity is indicated on each distribution. Note the logarithmic scale of the x-axis in panel (B). In both healthy dogs and dogs with liver pathology, the A allele is not associated with changes in AST activity.
(TIF)

S1 Table Blood tests analyzed. Standard reference intervals and intervals accepted for this study (see methods).
(PDF)

S2 Table Mean of ln(ALT) values and allele frequency of QTL by breed. Mean ln(ALT) values computed from chemistry panels of clinically healthy adult dogs (number of dogs in parentheses). Allele frequency (AF) of the derived A allele at the GPT locus for each breed based on genotype data from Shannon et al (number of genotyped dogs per breed in parentheses). Four-letter breed abbreviations as used in Fig 3.
(PDF)

S3 Table Correlation of aspartate aminotransferase (ALT) activity and *GPT* genotype with alanine aminotransferase (AST) activity in clinically healthy dogs and dogs with liver disease or injury. *GPT* SNP genotypes were assigned a numerical value (0 = G/G, 1 = A/G, 2 = A/A). Ln(AST) activity was positively correlated with ln(ALT) activity and was positively correlated with the number of derived A *GPT* alleles only for dogs with liver disease or injury.
(PDF)
