# Supplementary figures and images for: Genetic Mapping of Novel Loci Affecting Canine Blood Phenotypes
Source: PLoS One. 2015 Dec 18;10(12):e0145199. doi: 10.1371/journal.pone.0145199 (PMC4690602; doi:10.1371/journal.pone.0145199)

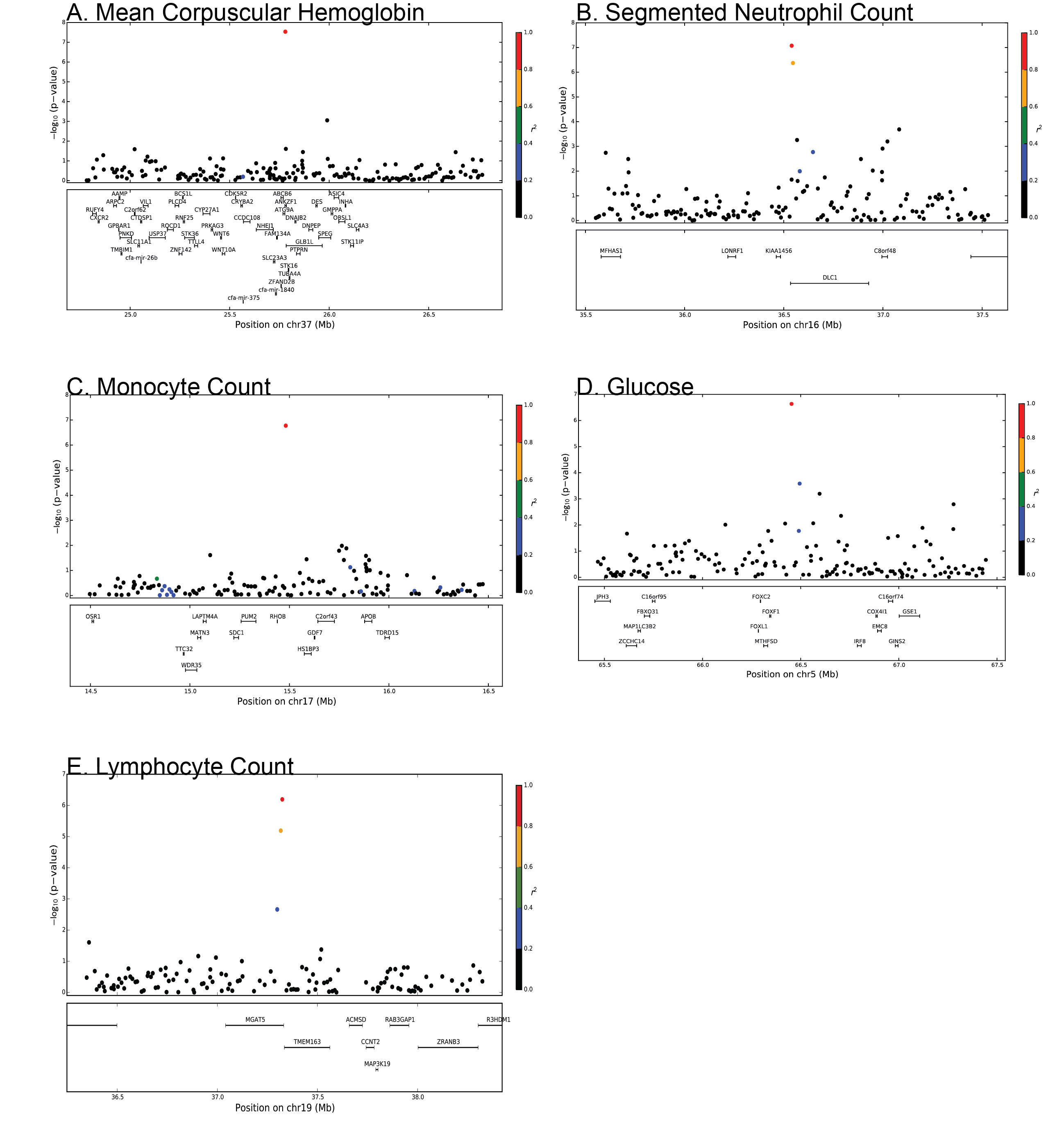

Supplement: S1 Fig — –log10P values of single marker associations with (A) Mean Corpuscular Hemoglobin, (B) Segmented Neutrophil Count, (C) Monocyte Count, (D) Glucose and (E) Lymphocyte Count. Circle color indicates level of linkage disequilibrium (r2) between each marker and the most associated SNP. The bottom panels show the relative position genes in the region. (TIF) [file pone.0145199.s001.tif]

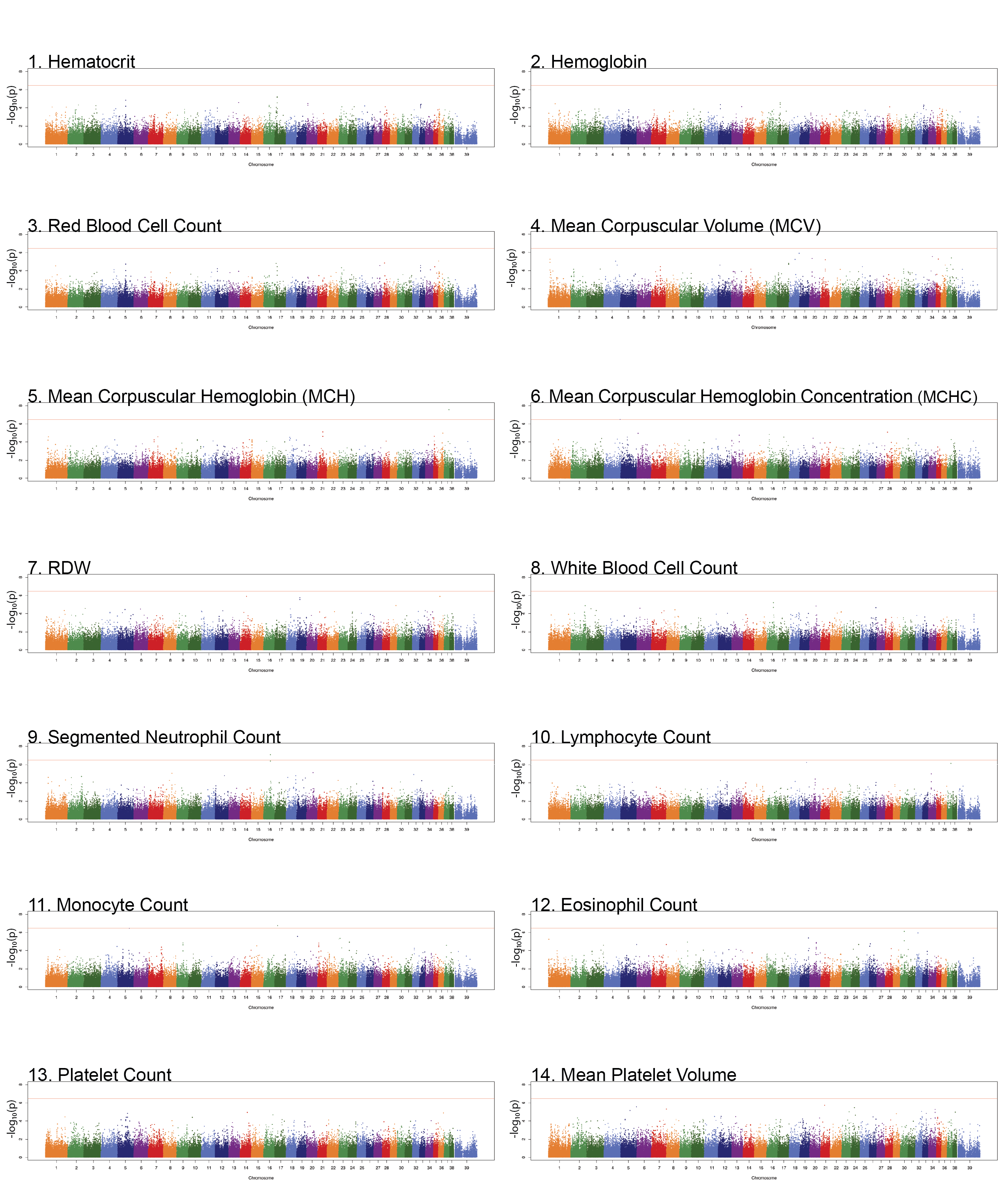

Supplement: S2 Fig — -log10P values of association between each SNP and each (transformed) phenotype. 1. Hematocrit, 2. Hemoglobin, 3. Red Blood Cell Count, 4. Mean Corpuscular Volume (MCV), 5. Mean Corpuscular Hemoglobin (MCH), 6. Mean Corpuscular Hemoglobin Concentration (MCHC), 7. Red Cell Distribution Width (RDW), 8. White Blood Cell Count, 9. Segmented Neutrophil Count, 10. (Square Root) Lymphocyte Count, 11. (Square Root) Monocyte Count, 12. (Square Root) Eosinophil Count 13. (Square Root) Platelet Count, 14. Mean Platelet Volume. (TIF) [file pone.0145199.s002.tif]

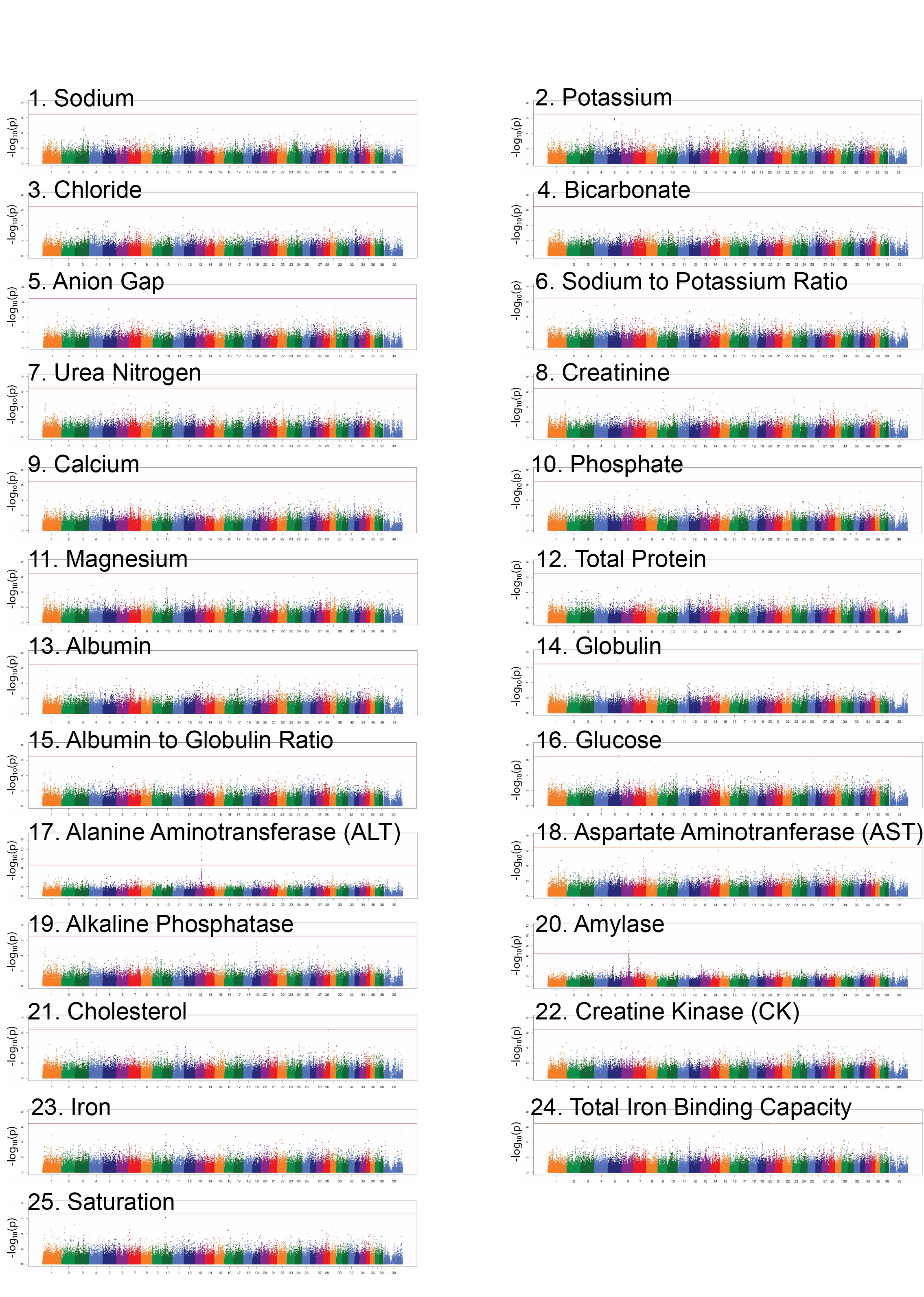

Supplement: S3 Fig — log10P values of association between each SNP and each (transformed) phenotype. 1. Sodium, 2. Potassium, 3. Chloride, 4. Bicarbonate, 5. Anion Gap, 6. Sodium to Potassium Ratio, 7. (Ln) Urea Nitrogen, 8. Creatinine, 9. Calcium, 10. (Square Root) Phosphate, 11. Magnesium, 12. Total Protein, 13. Albumin, 14. (Ln) Globulin, 15. Albumin to Globulin Ratio, 16. (Square Root) Glucose, 17. (Ln) Alanine Aminotransferase (ALT), 18. (Ln) Aspartate Aminotransferase (AST), 19. Alkaline Phosphatase, 20. (Square Root) Amylase, 21. Cholesterol, 22. (Ln) Creatine Kinase (CK), 23. Iron 24. Total Iron Binding Capacity, 25. (Ln) Percentage Transferrin Saturation (Saturation). (TIF) [file pone.0145199.s003.tif]

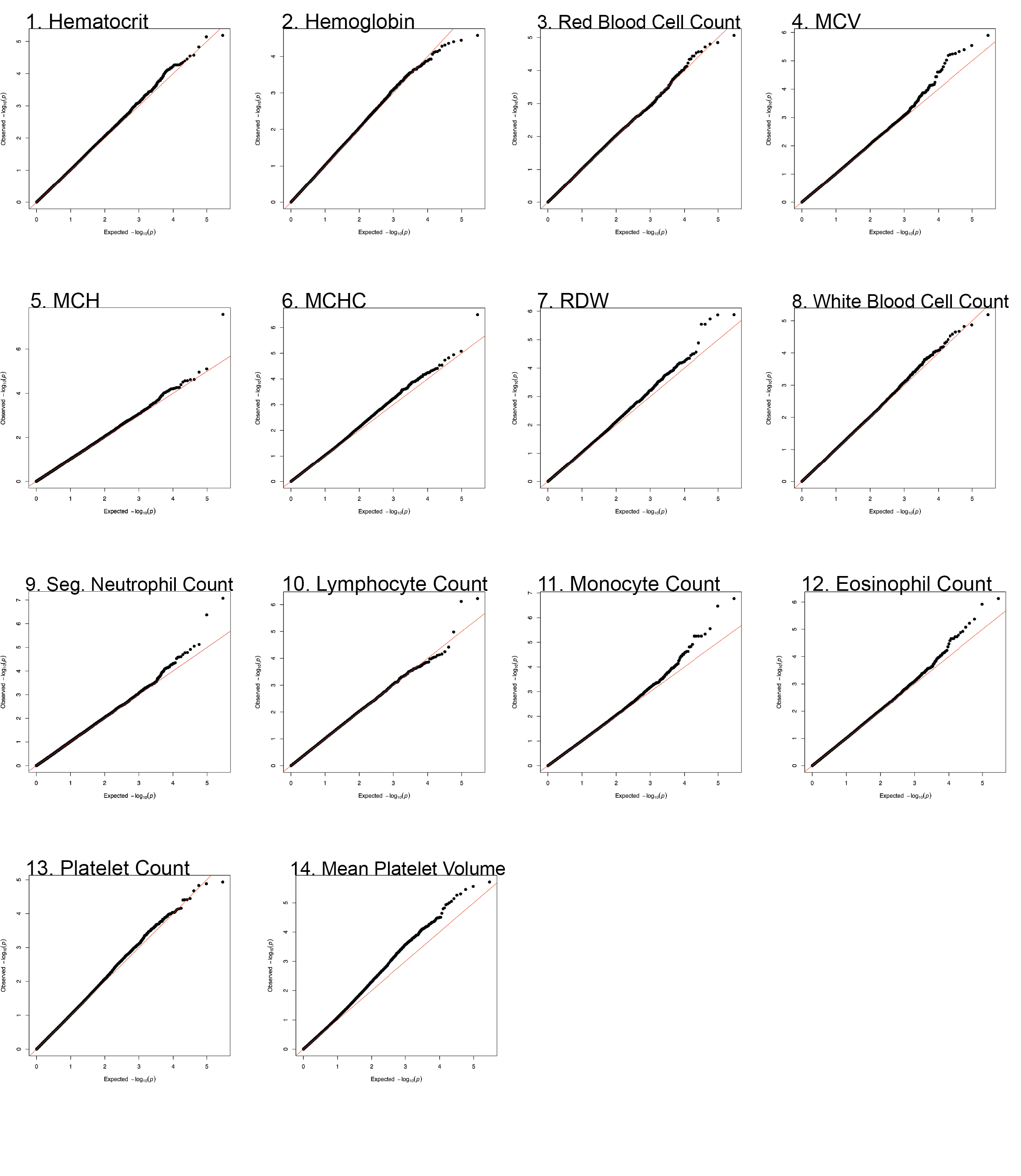

Supplement: S4 Fig — Quantile-quantile plots of observed versus expected association P-values. 1. Hematocrit, 2. Hemoglobin, 3. Red Blood Cell Count, 4. Mean Corpuscular Volume (MCV), 5. Mean Corpuscular Hemoglobin (MCH), 6. Mean Corpuscular Hemoglobin Concentration (MCHC), 7. Red Blood Cell Distribution Width (RDW), 8. White Blood Cell Count, 9. Segmented Neutrophils, 10. (Square Root) Lymphocyte Count, 11. (Square Root) Monocyte Count, 12. (Square Root) Eosinophil Count, 13. (Square Root) Platelet Count, 14. Mean Platelet Volume. (TIF) [file pone.0145199.s004.tif]

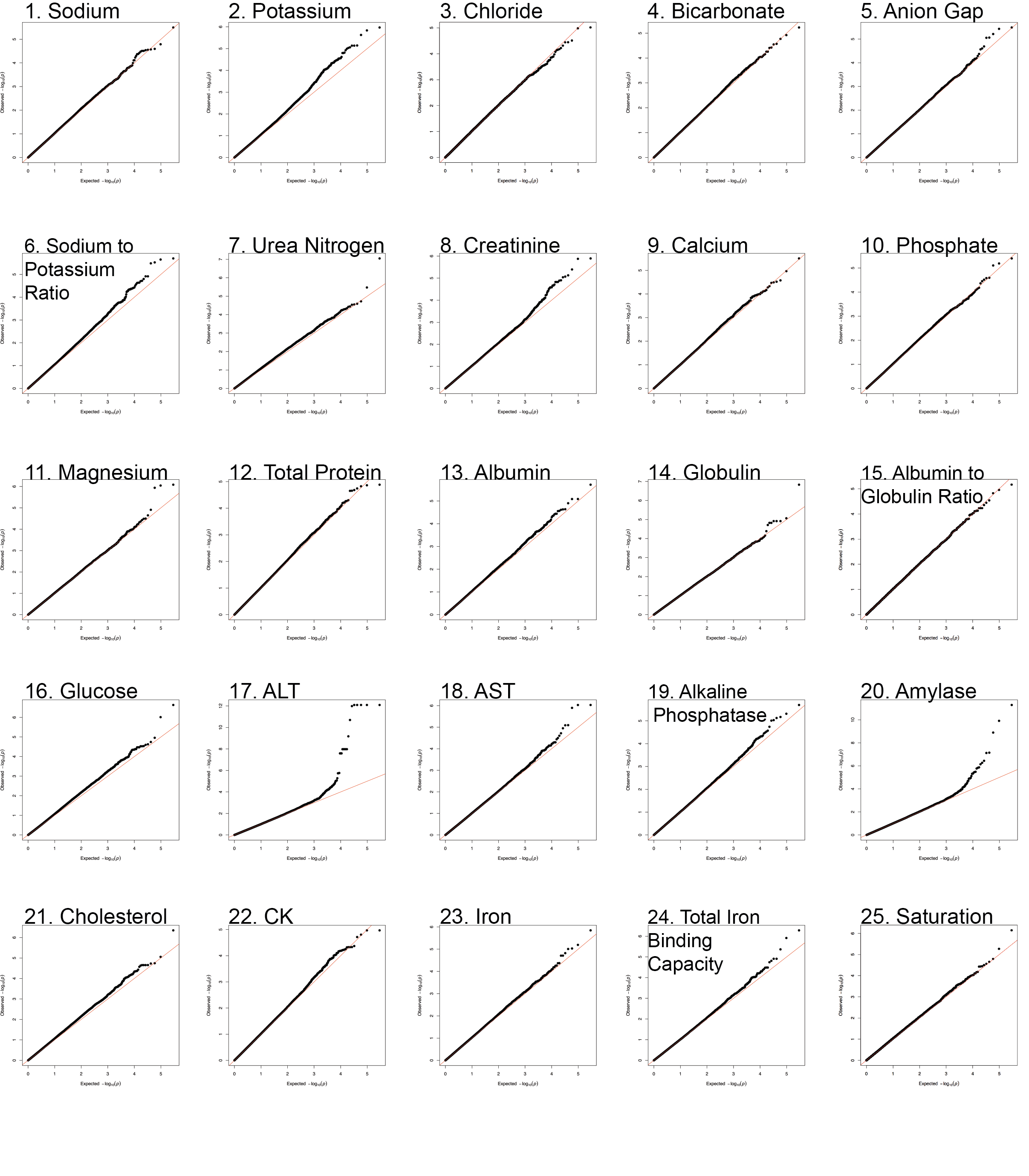

Supplement: S5 Fig — Quantile-quantile plots of observed versus expected association P- values. 1. Sodium, 2. Potassium, 3. Chloride, 4. Bicarbonate, 5. Anion Gap, 6. Sodium to Potassium Ratio, 7. Urea Nitrogen, 8. Creatinine, 9. Calcium, 10. (Ln) Phosphate, 11. (Square Root) Magnesium, 12. Total Protein, 13. Albumin, 14. (Ln) Globulin, 15. Albumin to Globulin Ratio, 16. (Square Root) Glucose, 17. (Ln) Alanine Aminotransferase (ALT), 18. (Ln) Aspartate Aminotransferase (AST), 19. Alkaline Phosphatase, 20. (Square Root) Amylase, 21. (Square root) Cholesterol, 22. (Ln) Creatine Kinase (CK), 23. (Square root) Iron 24. Total Iron Binding Capacity, 25. (Ln) Percentage Transferrin Saturation (Saturation). (TIF) [file pone.0145199.s005.tif]

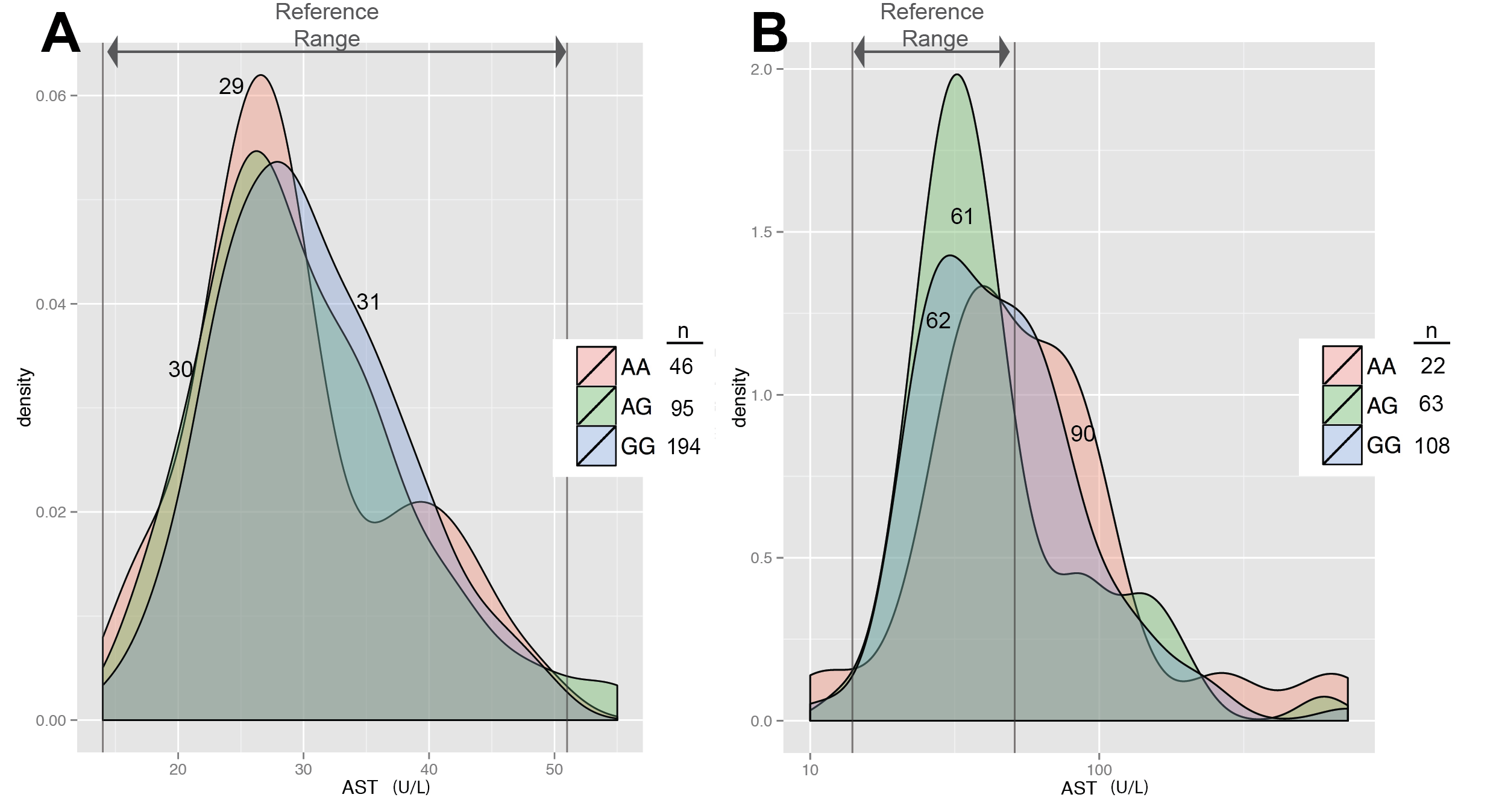

Supplement: S6 Fig — Distribution of AST activity by genotype of the most highly associated SNP for ALT on chromosome 13 in (A) healthy dogs and (B) dogs with liver disease or injury. Reference interval for AST activity shown with gray lines. Mean AST activity is indicated on each distribution. Note the logarithmic scale of the x-axis in panel (B). In both healthy dogs and dogs with liver pathology, the A allele is not associated with changes in AST activity. (TIF) [file pone.0145199.s006.tif]
