# Supplementary material for: Genetic Mapping of Novel Loci Affecting Canine Blood Phenotypes
Source: PLoS One. 2015 Dec 18;10(12):e0145199. doi: 10.1371/journal.pone.0145199 (PMC4690602; doi:10.1371/journal.pone.0145199)
Supplement: S1 Table — Standard reference intervals and intervals accepted for this study (see methods). (PDF) [file pone.0145199.s007.pdf]

Complete Blood Count Tests	Reference Interval	Accepted Interval
Hematocrit (%)	41-58	32-69
Hemoglobin (g/dL)	14.1-20.1	10.5-23.3
Red Blood Cell Count (mill/uL)	5.7-8.5	4.7-9.9
Mean Corpuscular Volume (fL)	64-76	60-81
Mean Corpuscular Hemoglobin (pg)	21-26	20-28
Mean Corpuscular Hemoglobin Conc (g/dL)	33-36	31-38.7
Red Blood Cell Distribution Width (%)	10.6-14.3	10.5-15.9
White Blood Cell Count (thou/uL)	5.7-14.2	3.3-19.9
Segmented Neutrophils (thou/uL)	2.7-9.4	2.1-17.3
Lymphocyte Count (thou/uL)	0.9-4.7	0.1-5.7
Monocyte Count (thou/uL)	0.1-1.3	0-2.7
Eosinophil Count (thou/uL)	0.1-2.1	0-2.9
Platelet Count (thou/uL)	186-545	103-734
Mean Platelet Volume (fL)	8.4-14.1	7.1-19.3

Clinical Chemistry Panel Tests	Reference Interval	Accepted Interval
Sodium (mEq/L)	142-150	141-155
Potassium (mEq/L)	3.8-5.4	3.4-5.5
Chloride (mEq/L)	105-116	103-122
Bicarbonate (mEq/L)	15-25	14-29
Anion Gap (mEq/L)	14-24	10-29
Sodium Potassium Ratio	Not Established	26-44
Urea Nitrogen (mg/dL)	10-32	5-48
Creatinine (mg/dL)	0.6-1.4	0.4-1.7
Calcium (mg/dL)	9.3-11.4	8.3-12.3
Phosphate (mg/dL)	2.9-5.2	1.6-8.1
Magnesium (mEq/L)	1.4-2.2	1.2-2.3
Total Protein (g/dL)	5.3-7	4.6-8
Albumin (g/dL)	3.1-4.2	2.8-4.6
Globulin (g/dL)	1.9-3.6	1.7-3.8
Albumin to Globulin Ratio	0.9-2.1	0.9-2.5
Glucose (mg/dL)	63-118	60-160
Alanine Aminotransferase (U/L)	20-98	10-278
Aspartate Aminotransferase (U/L)	14-51	10-126
Alkaline Phosphatase (U/L)	17-111	10-617
Amylase (U/L)	377-1220	154-1625
Cholesterol (mg/dL)	138-332	78-473
Creatine Kinase (U/L)	48-261	43-960
Iron (ug/dL)	78-214	17-329
Total Iron Binding Capacity (ug/dL)	270-496	162-606
Transferrin Saturation (%)	23-61	5-99
