# Supplementary material for: Genetic Mapping of Novel Loci Affecting Canine Blood Phenotypes
Source: PLoS One. 2015 Dec 18;10(12):e0145199. doi: 10.1371/journal.pone.0145199 (PMC4690602; doi:10.1371/journal.pone.0145199)
Supplement: S2 Table — Mean ln(ALT) values computed from chemistry panels of clinically healthy adult dogs (number of dogs in parentheses). Allele frequency (AF) of the derived A allele at the GPT locus for each breed based on genotype data from Shannon et al (number of genotyped dogs per breed in parentheses). Four-letter breed abbreviations as used in Fig 3. (PDF) [file pone.0145199.s008.pdf]

Breed	Abbr.	ln(ALT) (*N*)	AF (*N*)
Australian Shepherd	AUST	3.934 (207)	0.553 (38)
Basset Hound	BASS	3.959 (126)	0.275 (40)
Bernese Mountain Dog	BNMD	3.989 (92)	0.044 (68)
Bichon Frise	BICH	4.289 (93)	0.068 (44)
Border Terrier	BRTE	3.882 (20)	0.677 (68)
Boston Terrier	BOST	4.199 (107)	0.024 (42)
Boxer	BOXR	4.171 (234)	0.174 (344)
Cocker Spaniel	ECOC	3.880 (371)	0.453 (128)
Dachshund	DACH	4.120 (373)	0.475 (80)
Dalmatian	DALM	4.044 (22)	0.733 (30)
English Bulldog	ENGB	3.841 (103)	0.180 (50)
English Setter	ENGS	3.843 (32)	0.196 (194)
English Springer Spaniel	ENSS	3.953 (53)	0.538 (212)
French Bulldog	FRBU	4.033 (56)	0.111 (54)
German Shepherd	GERS	3.923 (644)	0.138 (566)
Golden Retriever	GOLD	3.827 (574)	0.634 (612)
Greyhound	GREY	4.141 (70)	0.094 (32)
Havanese	HAVA	4.225 (49)	0.352 (88)
Jack Russell Terrier	JACK	4.267 (285)	0.378 (74)
Labrador Retriever	LABR	4.136 (2192)	0.093 (1266)
Lhasa Apso	LHAS	4.047 (51)	0.100 (30)
Maltese	MALT	4.395 (228)	0.441 (170)
Mastiff Nos	MAST	3.830 (49)	0.045 (44)
Newfoundland	ELKH	3.752 (72)	0.637 (212)
Pembroke Welsh Corgi	PWCO	4.176 (32)	0.036 (28)
Pomeranian	POMR	4.295 (90)	0.500 (32)
Poodle Nos	POOD	4.167 (173)	0.324 (74)
Rottweiler	ROTT	3.755 (456)	0.969 (192)
Scottish Terrier	SCOT	4.429 (29)	0.114 (44)
Shetland Sheepdog	SHEL	4.042 (222)	0.365 (52)
Shih Tzu	STZU	4.160 (255)	0.067 (60)
Siberian Husky	SIBE	4.088 (89)	0.177 (34)
Tibetan Spaniel	TNSP	4.101 (22)	0.081 (62)
Toy Poodle	PDLT	4.101 (22)	0.500 (32)
Vizsla	VIZS	4.132 (35)	0.210 (186)
Weimaraner	WEIM	4.078 (103)	0.458 (72)
Yorkshire Terrier	YORK	3.977 (385)	0.597 (422)
