# Supplementary material for: Genetic Mapping of Novel Loci Affecting Canine Blood Phenotypes
Source: PLoS One. 2015 Dec 18;10(12):e0145199. doi: 10.1371/journal.pone.0145199 (PMC4690602; doi:10.1371/journal.pone.0145199)
Supplement: S3 Table — GPT SNP genotypes were assigned a numerical value (0 = G/G, 1 = A/G, 2 = A/A). Ln(AST) activity was positively correlated with ln(ALT) activity and was positively correlated with the number of derived A GPT alleles only for dogs with liver disease or injury. (PDF) [file pone.0145199.s009.pdf]

		ln(ALT)		*GPT* Genotype	
	**N**	**Effect Size**	***P* value**	**Effect Size**	***P* value**
**Clinically healthy**	**330**	**0.22**	**6.37×10^{-8}**	**0.03**	**0.181**
**Liver disease or injury**	**193**	**0.49**	**$<2 \times 10^{-16}$**	**0.17**	**0.002**
